# Identification of genes expressed in the hermaphrodite germ line of *C. elegans *using SAGE

**DOI:** 10.1186/1471-2164-10-213

**Published:** 2009-05-09

**Authors:** Xin Wang, Yongjun Zhao, Kim Wong, Peter Ehlers, Yuji Kohara, Steven J Jones, Marco A Marra, Robert A Holt, Donald G Moerman, Dave Hansen

**Affiliations:** 1 Department of Biological Sciences, University of Calgary, Calgary, Alberta T2N 1N4, Canada; 2 Canada's Michael Smith Genome Sciences Centre, British Columbia Cancer Agency, Vancouver, British Columbia V5Z 4S6, Canada; 3 Department of Mathematics and Statistics, University of Calgary, Calgary, Alberta T2N 1N4, Canada; 4 National Institute of Genetics, 1111 Yata, Mishima 411-8540, Japan; 5 Department of Zoology, University of British Columbia, Vancouver, British Columbia V6T 1Z3, Canada

## Abstract

**Background:**

Germ cells must progress through elaborate developmental stages from an undifferentiated germ cell to a fully differentiated gamete. Some of these stages include exiting mitosis and entering meiosis, progressing through the various stages of meiotic prophase, adopting either a male (sperm) or female (oocyte) fate, and completing meiosis. Additionally, many of the factors needed to drive embryogenesis are synthesized in the germ line. To increase our understanding of the genes that might be necessary for the formation and function of the germ line, we have constructed a SAGE library from hand dissected *C. elegans *hermaphrodite gonads.

**Results:**

We found that 4699 genes, roughly 21% of all known *C. elegans *genes, are expressed in the adult hermaphrodite germ line. Ribosomal genes are highly expressed in the germ line; roughly four fold above their expression levels in the soma. We further found that 1063 of the germline-expressed genes have enriched expression in the germ line as compared to the soma. A comparison of these 1063 germline-enriched genes with a similar list of genes prepared using microarrays revealed an overlap of 460 genes, mutually reinforcing the two lists. Additionally, we identified 603 germline-enriched genes, supported by *in situ *expression data, which were not previously identified. We also found >4 fold enrichment for RNA binding proteins in the germ line as compared to the soma.

**Conclusion:**

Using multiple technological platforms provides a more complete picture of global gene expression patterns. Genes involved in RNA metabolism are expressed at a significantly higher level in the germ line than the soma, suggesting a stronger reliance on RNA metabolism for control of the expression of genes in the germ line. Additionally, the number and expression level of germ line expressed genes on the X chromosome is lower than expected based on a random distribution.

## Background

Germ cells follow an elaborate developmental program to produce fully differentiated gametes. In most animals, germ cells first proliferate to generate a pool of cells, some of which cease proliferating and enter into meiotic prophase, progress through the many stages of meiosis and finally differentiate into sperm or oocytes. Throughout the entire process of gamete formation, genes involved in differentiation of the soma must be repressed, and genes necessary for progression through meiotic prophase, gamete formation and germline sex determination must be expressed [1]. Additionally, many of the factors needed for embryogenesis are produced in the germ line. Much of our understanding of the many steps involved in germline development and gamete formation has come from study of genetically tractable model organisms, such as *Drosophila *and *C. elegans*. The purpose of this study is to identify many of the genes involved in the proper function of the *C. elegans *hermaphrodite germ line.

**Figure 1:**
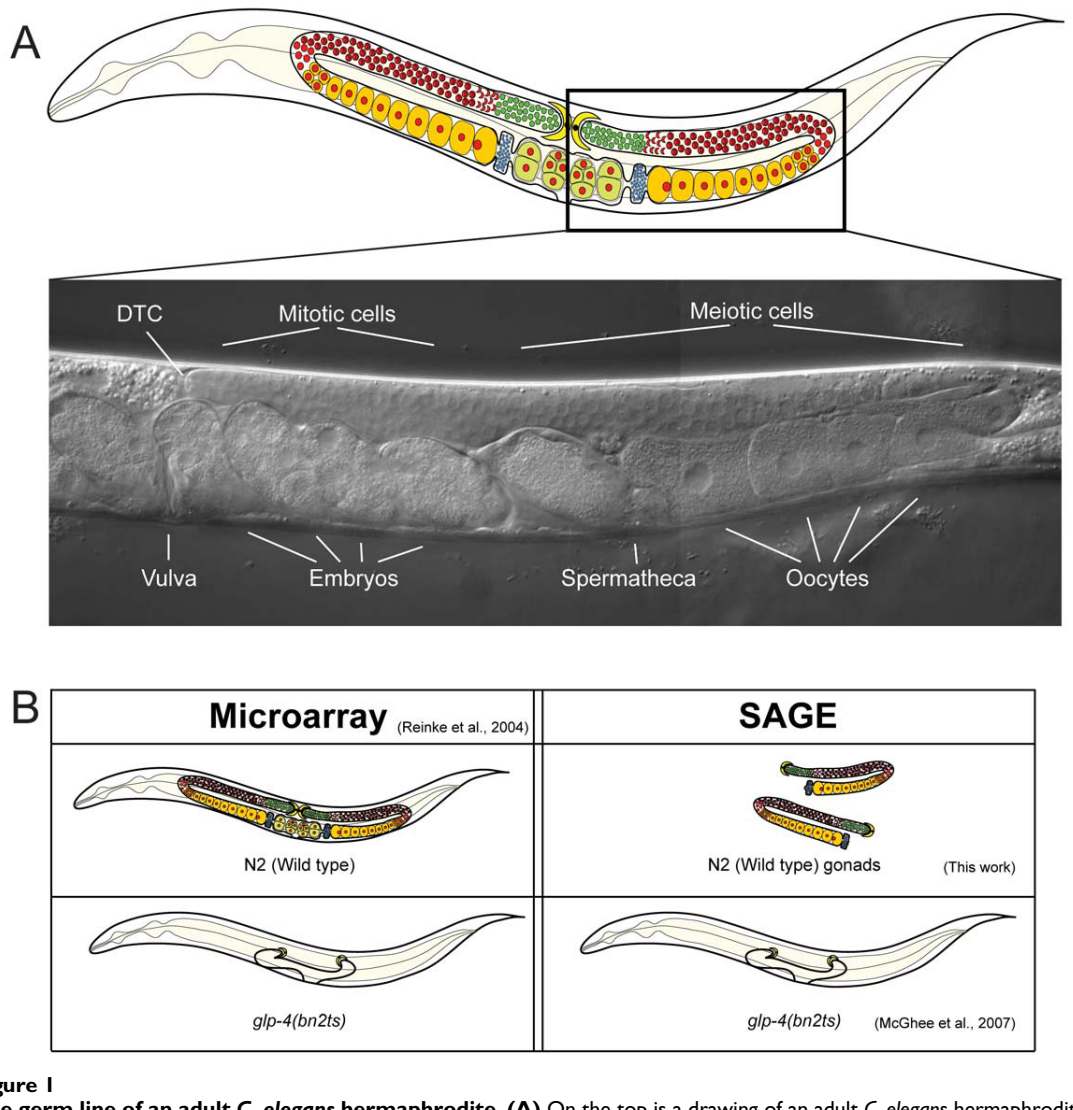
**The germ line of an adult *C. elegans *hermaphrodite**. **(A) **On the top is a drawing of an adult *C. elegans *hermaphrodite emphasizing the cells of the germ line. The gonad consists of two reflexed arms that meet at a common uterus. At the very distal end of each arm is the somatic distal tip cell (DTC; yellow). Germ cells near the DTC are proliferative (green). As cells move proximally, towards the uterus, they enter meiotic prophase (red). The first cells to differentiate are sperm (blue), which are stored in the spermatheca. As oocytes (orange) pass through the spermatheca, they become fertilized and begin embryogenesis (green). Below the diagram is a DIC image of one gonad arm in an adult hermaphrodite. **(B) **Illustrated is a summary of the tissues used to obtain mRNA for the germline microarray analysis [11], the soma SAGE library [24] and the germline SAGE library (this work). In the microarray analysis mRNA obtained from wild-type (N2) worms was compared to mRNA obtained from *glp-4(bn2ts) *worms. Germ cells in *glp-4(bn2ts) *animals grown at the restrictive temperature arrest in mitosis such that only ~12 germ cells are present [41]. The somatic gonad is still present, but the gonad arms do not reflex back. mRNA for the construction of the germline SAGE library was obtained from ~150 hand dissected gonad arms. The gonad arms were dissected away from the body of the animal at or near the spermatheca. The soma SAGE library was constructed from mRNA isolated from *glp-4(bn2ts) *animals grown at the restrictive temperature.

The gonad in the *C. elegans *hermaphrodite consists of two U-shaped tubes that meet at a common uterus (Figure 1) [2]. The gonad is completely enclosed by a basement membrane. Along the length of the gonad, and within the basement membrane, are five pairs of somatic sheath cells that provide structure to the gonad, as well as provide some reproductive functions [2-4]. The end of each tube furthest, or most distal, from the uterus is capped by a somatic cell called the distal tip cell (DTC). Most of the nuclei within the gonad are only partially enclosed by membranes; i.e., they are syncitial. We will refer to the nuclei, their surrounding cytoplasm, and partially enclosing membranes as cells. Germ cells closest to the DTC are proliferative. As the proliferative germ cells divide, they move away from the DTC, towards the uterus. Once they are approximately 20 cell diameters from the DTC, they show the first signs of entering into meiotic prophase [5,6]. These early meiotic cells have a crescent shaped nuclear morphology and are within a region of the gonad referred to as the transition zone [5,7]. As cells continue to move proximally, towards the uterus, they progress through the various stages of meiotic prophase. In the hermaphrodite, the first ~40 germ cells in each gonad arm differentiate as ~160 spermatocytes, which are stored in the spermatheca [2]. All subsequent germ cells differentiate as oocytes, which undergo ovulation and enter the spermatheca, where they are fertilized by the sperm. The zygote then moves into the uterus and undergoes early embryonic development before being expelled into the environment through the vulva [2]. While there are only 959 somatic cells in the adult hermaphrodite, each gonad arm generates about 1000 cells [8,9]. Somatic cells do not divide in the adult hermaphrodite; however, germ cells continue to proliferate and form gametes throughout much of the adult life of the animal [2,9]. Therefore, a substantial amount of energy and resources in the adult hermaphrodite are spent on producing gametes.

Genetic screens, both forward and reverse, have identified many of the genes and genetic pathways involved in generating gametes [10]. However, we are still far from fully uncovering the underlying biological complexity of gamete formation. Large-scale microarray studies have greatly assisted in determining the genes involved in germline development and gamete formation by identifying mRNA transcripts that are enriched in the germ line [11,12]. As part of these studies, transcripts in intact animals were compared to animals that lacked a germ line due to genetic mutation. In L4 and adult hermaphrodites, 3144 genes were demonstrated to have germline-enriched expression. The generation of this data set, as well as data sets of male germ cells, female germ cells and staged larvae, have assisted in our general understanding of the genes involved in gamete formation, as well as provided a starting point for more detailed analyses of the specific roles these genes play in the germ line [7,13-21]. However, all genome analysis technological platforms have inherent advantages and disadvantages [22]. Therefore, using multiple technological platforms provides a more complete view of the transcription profile of the tissue or animal being studied, as well as provide increased confidence of the overlapping data. It also provides an opportunity to analyze the strengths and limitations of each platform.

Here we describe our analysis of germline transcription in the adult hermaphrodite germ line using Serial Analysis of Gene Expression (SAGE) [23]. This analysis adds to previous microarray analyses by identifying genes transcribed in the germ line independent of their level of expression in the soma [11,12]. Furthermore, we use the SAGE data as a measure of relative expression levels between genes to identify the genes and gene classes whose mRNA is most abundant in the germ line. Finally, by comparing our data with previously published soma SAGE data [24], we are able to identify genes that are enriched in the germ line, which allows for a comparison of the SAGE and microarray platforms [11].

## Results

### Production and overview of the germline SAGE library

We constructed a SAGE library from ~150 hand dissected *C. elegans *hermaphrodite gonads to identify genes that are transcribed in the germ line. The germline SAGE library identified 92,007 tags, which was normalized to 100,000 tags for further analysis. While important previous studies have identified transcribed genes that are expressed at a higher level in the germ line than the soma [11,12], our analysis identifies genes that are expressed in the germ line irrespective of their level of soma expression. This provides a broader picture of genes involved in germline function, independent of the roles the genes may have in the soma. Additionally, since the number of tags per gene is quantified in SAGE, we are able to consider the relative levels of expression between genes.

We dissected the gonad arms away from the soma of the animals at, or close to, the spermatheca (see Methods) (Figure 1). Therefore, the cell types in the dissected gonad arms include the proliferative germ cells at the distal end of the gonad, germ cells at various stages of meiotic prophase, oocytes, and perhaps some sperm. The dissected gonads also contain some cells of the somatic gonad, including the distal tip cell, sheath cells, and at least some of the 24 cells that form the spermatheca.

**Figure 2:**
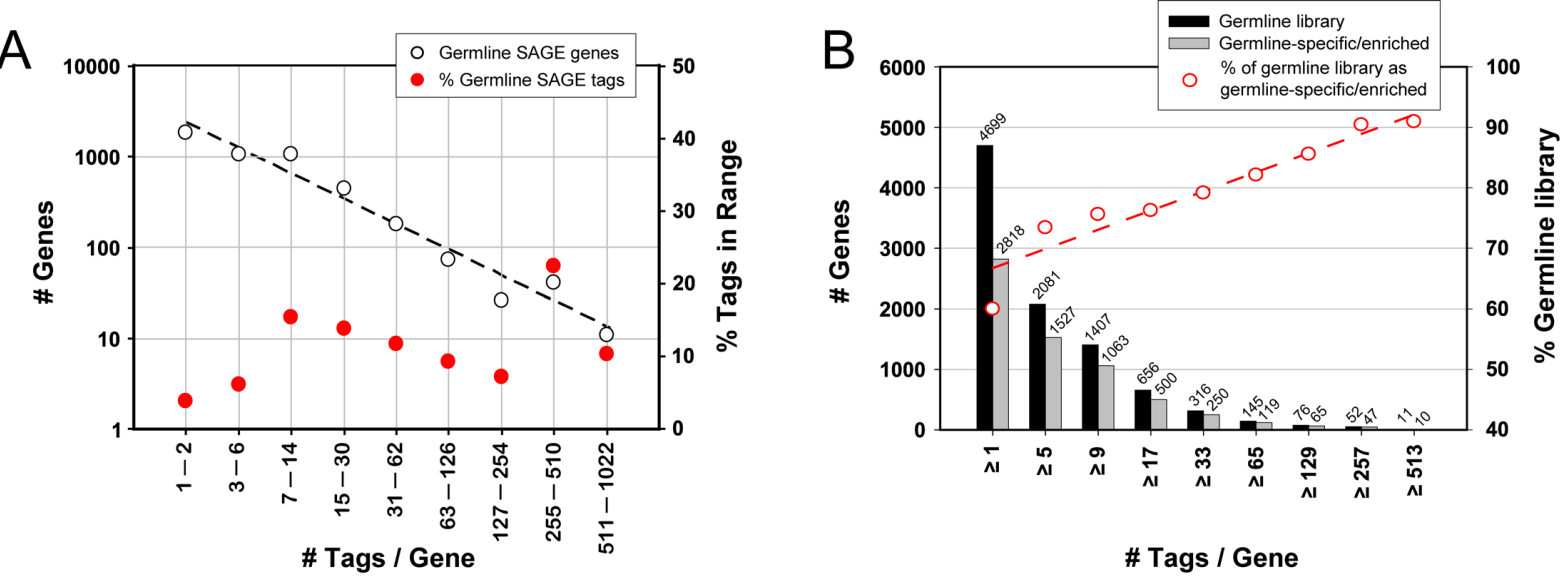
**Distribution of transcripts in the germline SAGE library and the germline-specific/enriched dataset identified**. **(A) **The distribution of tag counts in the germline SAGE library obeys a power law. More genes were identified as being expressed at low transcript levels, whereas significantly fewer genes are expressed at modest to high transcript levels. However, considerably higher numbers of tags are identified by the small subset of genes expressed at relatively high levels. Only 52 genes were identified to have a tag counts > 254, whereas they account for ~33% the total tags identified in the germline library. **(B) **The portion of the germline library identified to be germline-specific/enriched increases with increasing tag counts. Tag count ≥ 9 was chosen as a cut off to increase the confidence of the germline-specific/enriched gene set. With tag count ≥ 9, 1063 out of 1407 (~75%) of the germline library genes were identified to be germline-specific or germline-enriched.

The analysis of the germline SAGE library identified a total of 4699 genes with associated SAGE tags (Additional file 1), suggesting that these genes are transcribed in the germ line (see Methods for a description of the criteria used to determine genes associated with SAGE tags). These expressed genes correspond to approximately 21% of the total predicted genes in the *C. elegans *genome. The germline-expressed genes have a wide range of expression levels based on the number of tags per gene, with a low of one tag per gene to a high of 916 tags per gene (Figure 2; Additional file 1). The number of genes within a range of SAGE tags per gene obeys a power law; that is, that the logarithm of the number of tags per gene has a linear relationship to the logarithm of the number of genes that have a similar number of tags (Figure 2a). Therefore, the number of genes with a given expression level decreases as the level of expression increases (Figure 2). Other *C. elegans *SAGE libraries have also shown a power law relationship between the expression level of a group of genes, and the number of genes within the group [24].

### Genes highly represented in SAGE library

To better understand the functions of the genes with the most abundant transcript levels in the *C. elegans *hermaphrodite gonad, we analyzed the genes with the highest tag counts. So as to analyze a manageable list of genes, we analyzed genes with tag counts greater than 60, which resulted in a list of 157 genes (Additional file 2). To first corroborate our identification of these genes as being expressed in the germ line, we categorized their expression patterns using the NEXTDB large scale *in situ *database, which provides pictures of mRNA *in situ *staining patterns at various stages of development for many genes in the genome [25]. 124 of the 157 genes are included in the NEXTDB database and have discernable expression patterns (Additional file 2). All of the 124 genes showed expression in the germ line, 18% with exclusive germline expression, 63% with enriched germline expression and 19% with similar expression in the germ line and soma (Additional file 2). Therefore, the NEXTDB mRNA *in situ *expression patterns are consistent with our identification of these genes as being transcribed in the germ line. The functions of most of these genes have been analyzed, at a gross level, in large-scale RNA interference (RNAi) screens [26,27]. The majority of the genes (83%) have phenotypes consistent with a germline function, such as germ cell proliferation abnormal (Gpro), gonad development abnormal (Gdb), sterile (Ste), sterile progeny (Stp) and abnormal embryogenesis (Emb) (Additional file 2) (This is statistically significant, p < 0.001, as when we analyzed all genes in the genome tested by RNAi, only 15.5% have these germline phenotypes). Some of the genes not showing a germline phenotype may still have a germline function, but the phenotype may have been too subtle to be detected in large-scale screens, or they may function redundantly with another gene(s). It is also possible that some genes that are transcribed in the germ line do not have germline functions (see Discussion). The NEXTDB expression patterns and RNAi phenotypes both support our detection of these genes as germline-expressed.

**Figure 3:**
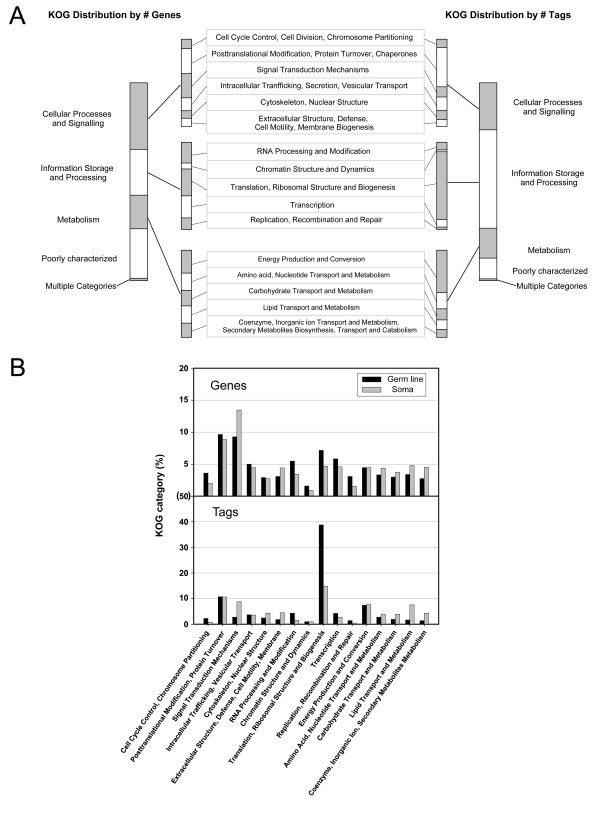
**KOG classification of the germline SAGE library**. **(A) **KOG distribution of the germline library based on either the number of genes or the number of tags. A total of 3555 genes can be assigned a KOG classification in the germline SAGE library. "Information Storage and Processing" is over-represented in distribution by the number of tags, and this over-representation is mainly contributed by the subcategory "Translation, Ribosomal Structure and Biogenesis". The over-representation of "Information Storage and Processing" is not observed in the soma library (data not shown). Overall, the KOG distribution by the number of genes is highly similar between the germline library and the soma library; however, some categories or sub-categories do show some enrichment in the germ line or soma, the most striking of which are described in the text (data not shown). **(B) **Shown are the percentages of the KOG sub-categories with respect to the total number of genes (upper panel) or the total number of tags (lower panel) assigned with KOGs.

To gain a general picture of the functions of the 157 genes with the highest expression in the germ line, as well as the functions of all 4699 genes with SAGE tags in the germline library, genes were annotated with KOG (eukaryotic orthologous groups) descriptions and classified into KOG categories and sub-categories (Additional file 2; Figure 3a) [28,29]. For all germline-expressed genes, we determined the number of genes in each KOG category, as well as the number of tags in each category (Figure 3a). Of all of the genes identified in the germline SAGE library, ~75% have associated KOG terms. However, ~90% of tags identified correspond to genes with associated KOG terms; therefore, many of the genes lacking a KOG term have relatively low expression levels. The KOG category with by far the most germline-expressed genes is *Information storage and processing*, particularly its sub-category *Translation, ribosomal structure and biogenesis *[compared to soma SAGE library (see below) there is a significant enrichment of this sub-category in the germ line, p < 0.001] (Figure 3); 66 of the 157 most highly expressed genes fall within this sub-category (Additional file 2). While most of the genes in this sub-category encode ribosomal proteins, also included are some genes encoding translational regulators; *puf-3*, *puf-5 *and *puf-11 *each encode proteins homologous to the *Drosophila *Pumilio. The Puf (Pumilio and *fbf*) family of proteins consists of similar RNA binding proteins that have been shown to control many cellular and developmental processes in many tissues, including the *C. elegans *germ line [30-36].

Among the 157 genes with the highest tag counts in the germ line are a number of genes of the ubiquitin/proteasome protein degradation pathway (Additional file 2). While proteasome mediated degradation of proteins was once thought to be primarily involved in the degradation of misfolded or damaged proteins, increasing evidence has shown that proteasome mediated degradation is an important regulatory mechanism in the development and function of cells and tissues [37]. In the *C. elegans *germ line, the proteasome has been implicated in the regulation of germline sex determination and the proliferation vs. meiotic entry decision in the germline stem cells [38-40].

Many of the 157 genes most highly expressed in the germ line are often referred to as 'house-keeping' genes, or genes that are necessary for the general function of virtually any cell, including genes involved in mitochondrial function, ribosome structure and other cellular processes. To determine if the number of genes and their expression levels observed in the germ line are typical of the soma, we first determined the number of genes and number of SAGE tags for each KOG category and sub-category for the entire germline SAGE library (Figure 3a). We then compared the number of genes and number of tags in each KOG sub-category in the germline SAGE library with the number of genes and tags in each sub-category from a soma SAGE library (Figure 3b). A soma *C. elegans *SAGE library has already been published, which was generated from worms lacking a germ line due to the *glp-4(bn2ts) *temperature sensitive mutation [24]. Germ cells in *glp-4(bn2ts) *worms, grown at the restrictive temperature, arrest early in mitosis allowing only ~12 germline nuclei to be formed per gonad arm; however, the soma in *glp-4(bn2ts) *animals appears wild type [41]. Therefore, the soma library was generated from virtually all tissues not used in the generation of our germline SAGE library; however, some cells of the somatic gonad are common to both libraries. In the germline SAGE library there is a modest increase in the number of expressed genes in the *Translation, ribosomal structure and biogenesis *KOG sub-category as compared to the soma (Figure 3b). However, when gene expression levels are taken into account, based on tag number, there is a dramatic increase in the *Translation, ribosomal structure and biogenesis *sub-category in the germline SAGE library as compared to the soma library (p < 0.001; Figure 3b). This increase in tag numbers in the germline SAGE library is primarily due to the large number of tags associated with ribosomal genes. Among the 87 ribosomal protein encoding genes in the *C. elegans *genome [42], 53 are identified by SAGE to be highly expressed (tag count >60) in the germ line (23 ribosomal small subunits and 30 large subunits; Additional file 2). The expression levels for the ribosomal genes are strikingly high, as they produce ~27% of all tags in the germline SAGE library (Figure 3a). The high level of expression of these ribosomal genes suggests that ribosome biogenesis is an essential component to maintain normal germline function and development (see Discussion). Supporting this idea, nearly all of the ribosomal genes show RNAi phenotypes consistent with a germline function, such as embryonic lethality/developmental defect and sterility [26,27] (Additional file 2).

Other KOG sub-categories that have a higher percentage of tag counts (p < 0.001), suggesting higher expression levels, in the germ line than the soma are; (1) *Cell cycle control, chromosome partitioning*, (2) *Replication, recombination and repair*, (3) *Transcription*, and (4) *RNA processing and modification*. It is not surprising that genes in the first two sub-categories are expressed at a higher level in the germ line. Since both the soma and germline libraries were generated from adult tissue, and since the germ line is the only tissue with dividing cells in the adult, it is logical that these two sub-categories, which deal with the division of cells, have a higher level of expression in the germ line than the soma. The third and fourth sub-categories listed above have to do with the transcription of mRNA and its modification. It seems reasonable that if more translation occurs in the germ line than other tissues, based on the high level of expression of ribosomal genes, more mRNA would need to be made and processed.

All other KOG sub-categories have either similar expression levels in the soma and germ line, or have higher expression levels in the soma. Perhaps the two most striking examples of KOG sub-categories with higher expression levels in the soma than germ line are *Lipid transport and metabolism *and *Signal transduction mechanisms *(p < 0.001). The intestine is likely the tissue in which most energy production occurs, and many genes involved in lipid metabolism are enriched in the intestine [24]; therefore, it is logical that expression levels are higher in the soma than germ line. The greater than two-fold increase in expression in genes involved in *Signal transduction mechanisms *suggests that the soma relies more heavily on signaling for proper function.

### Genes encoding RNA binding proteins are expressed at a higher level in the germ line

Many RNA binding proteins have essential functions in the germ line of *C. elegans*, with RNA metabolism being a predominant mechanism for the control of gene expression in this tissue [43,44]. It has recently been suggested that the expression of proteins in the *C. elegans *germ line relies primarily upon the control of translation or mRNA stability regulated through the 3'UTR [45]. In order to determine if RNA regulators are expressed at a higher level in the germ line than the soma, we identified a list of 319 RNA binding proteins (See Methods; Additional file 3). We found that 190 of these genes are expressed in the germ line, based on the presence of one or more tags in the germline SAGE library, while only 131 of these genes are expressed in the soma. Taking into account the number of tags found in each library, we found that transcripts of these RNA binding proteins are expressed >4 fold in the germ line than the soma; 3267 tags were identified in the germline SAGE library, whereas only 775 were identified in the soma library (Fisher's exact p-value < 0.001). The higher expression level in the germ line is not due to one or few genes being expressed at much higher levels in the germ line; rather, most genes are expressed at a higher level in the germ line as compared to the soma (Additional file 4). The higher level of expression of genes encoding RNA binding proteins supports the model of the expression of genes in the germ line relying more heavily upon RNA metabolism for control.

### Identification of germline-enriched and germline-specific genes

The germline SAGE library allows us to identify genes transcribed in the germ line, as well as their relative expression levels, irrespective of their expression in the soma. Previous studies of germline expression using microarrays have produced valuable lists of genes whose transcripts are enriched in the germ line [11,12]. Genes that are expressed at a higher level in the germ line than the soma may have roles that are more specific to germline function. As mentioned above, a SAGE library has been generated from somatic tissues of adult hermaphrodites [24]. By comparing the expression levels (tag counts) of genes in our germline SAGE library with the expression levels of the same genes in the soma, as determined by the soma SAGE library, we can identify genes whose transcription is enriched in the germ line. This comparison is analogous to the microarray analyses that identified germline-enriched genes, in which animals with a germ line were compared to animals lacking a germ line (Figure 1b) [11,12]. All technological platforms, including SAGE and microarrays, have inherent strengths and weaknesses [22]; therefore, identifying germline-enriched genes using a different technological platform (SAGE instead of microarray), may allow for the identification of additional germline-enriched genes. Additionally, SAGE will help to validate many of the genes identified by microarray as being germline-enriched. Finally, an analysis of the lists of germline-enriched genes obtained by microarray and SAGE will help to uncover some of the inherent strengths and weaknesses of each technological platform.

Before we describe the comparison between the germline and soma SAGE libraries, it is important to emphasize that these two libraries were not made at the same time and differ somewhat in their construction, which complicates their comparison and the interpretation of results (see Methods). The primary difference is that the soma library was made using short SAGE, while the germline library was made using long SAGE. 'Short' and 'long' refers to the size of the SAGE tag produced upon restriction digestion [46]. Therefore, the soma and germline SAGE libraries differ somewhat in the genes that have associated unique SAGE tags. Genes that do not have SAGE tags in one or both of the libraries were not included in the comparison (see Methods). Other differences between the germline and soma SAGE libraries include the genotypes of the animals; the germline library was constructed from wild-type worms (N2), while the soma library was constructed from *glp-4(bn2) *worms [24], which caused them to lack germline tissue. However, these were the same genotypes that were used in the microarray analysis [11]. As a final difference, the wild-type worms for the germline SAGE library were grown at 20°, while the *glp-4(bn2ts) *worms were shifted from 15° to 25°, causing the animals to have severely reduced germ cell number [41]. While these differences in library construction may have an effect on the expression of some genes, independent of the inherent differences in germline and soma expression levels, the microarray data [11] and large scale *in situ *expression data (NEXTDB) [25] provide excellent tools to help determine the extent the differences in library construction may have on interpretation.

**Figure 4:**
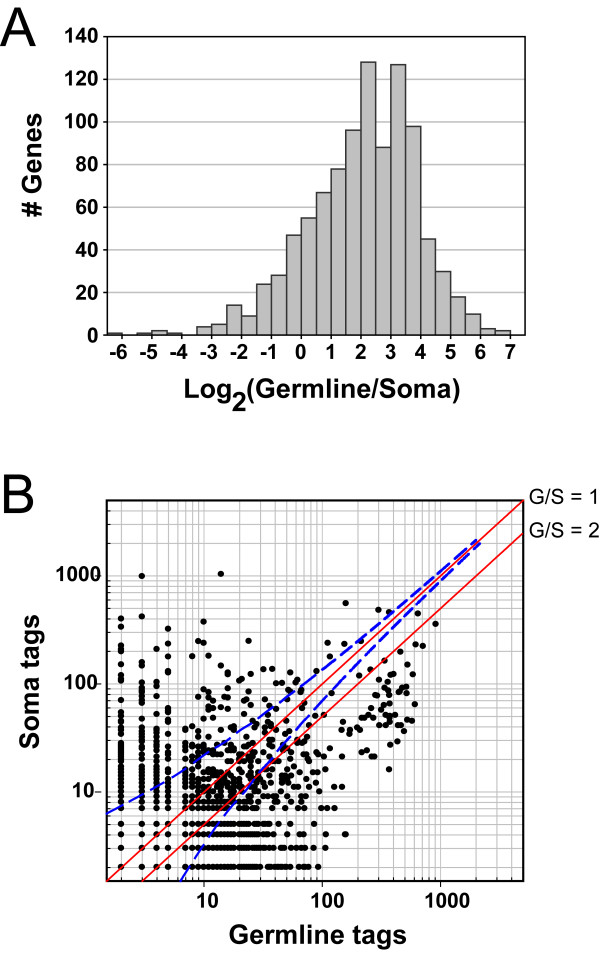
**Distribution of transcript levels between the germline and the soma**. **(A) **Histogram showing the distribution of the Germline/Soma tag count ratio for those common genes between the germline library and the soma library. The data correspond to 981 genes with germline tag counts ≥ 9 and soma tag counts ≥ 1. ~75% of these appear to have the Germline/Soma tag count ratio ≥ 2. **(B) **Scatter plot showing the distribution of tag counts for those common genes between the germline library and the soma library. The data represent 1552 genes with tag counts ≥ 2 in both the germline library and the soma library. Data points for genes that are expressed at the same level in the germ line and soma should fall along the mid-diagonal with a G/S tag ratio of 1, while genes that have enriched germline expression should be to the right of the diagonal with a G/S ≥ 2. The dashed blue line provides a measurement of sampling confidence based on the statistical analysis of [47]. Given a particular number of tags generated for a gene in the soma library, there is a 99% chance that the number of tags generated in the germline library for the same gene will lie to the left of the dashed blue line. In the set of germline-enriched genes that were selected, there are 41 genes below this 99% confident line; however, 19 out of the 23 genes that have a discrete *in situ *expression pattern in the NEXTDB appear to be germline-enriched.

To obtain a list of germline-enriched genes, we compared the number of tags in the germline SAGE library with the number of tags in the soma SAGE library for each gene (Figure 4) [47]. 87% of genes that were identified in the germline SAGE library show a germline/soma tag ratio ≥ 1, and 75% show a germline/soma ratio ≥ 2 (Figure 4a). Since differences in tag counts for genes with low numbers of tags are less likely to be statistically significant, we only analyzed genes with a tag count ≥ 9 in the germline SAGE library (Figure 4b). By using this cut-off, we remove the majority of genes that fail to reach the p < 0.01 confidence level (Figure 4b). This cut-off was also used in a previously published comparison of SAGE libraries [24]. We classified genes with a two-fold or greater increase in the number of SAGE tags in the germ line compared to soma as "germline-enriched", while those with ≥ 9 tags in the germline library and no tags in the soma library as "germline-specific" (see Methods). Using these criteria, we identified 733 genes that are germline-enriched and 330 genes that are germline-specific, for a total of 1063 genes that are either specifically expressed or enriched in the germ line (Additional file 5). The proportion of germline-expressed genes that are specific/enriched in the germ line is higher for genes with higher expression levels, based on tag counts, than for genes with lower expression levels (Figure 2b). Since germline-specific/enriched genes are expressed at a higher level in the germ line than soma, they may have functions that are more specific to germline function. The functions of many of these genes, based on KOG classification, are in keeping with the tremendous amount of cell division and tissue generation that occurs in the rapid formation of gametes (Additional file 5; see Discussion).

### Comparison of SAGE and Microarray data

**Figure 5:**
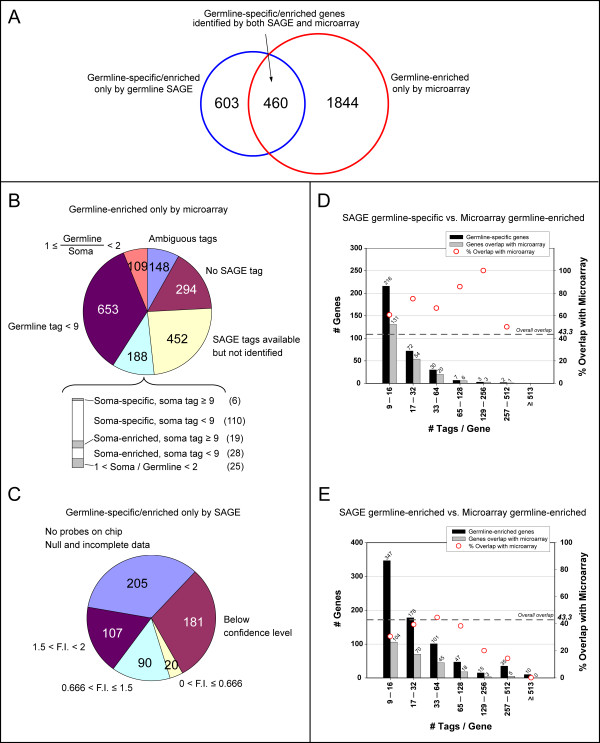
**Comparison of the germline-specific/enriched genes identified by SAGE with the germline-enriched genes identified by the microarray**. **(A) **Overview of the comparison between SAGE and microarray. Germline-specific/enriched SAGE data were generated by selecting genes that are identified only by the germline library (germline-specific) and genes that have a 2-fold enrichment of the tag counts in the germline library over the soma library (germline-enriched). Genes were further selected to have a germline tag count ≥ 9. Data correspond to 330 germline-specific genes and 733 germline-enriched genes. Microarray data used were the adult data, with *wt/glp-4 *F.I. ≥ 2 (99% confidence >0). **(B) **SAGE data for the germline-enriched genes identified only by microarray. 'Ambiguous genes' are genes that were not identified as germline-specific due to having an ambiguous tag in the soma (Short-SAGE) library, or that have an ambiguous Long-SAGE tag (Germline SAGE library). 'No SAGE tags' do not have an assigned Long and/or Short-SAGE tag. 'SAGE tags available but not identified' are genes that do have an associated Long-SAGE tag and Short-SAGE tag, and the tags are not ambiguous, but no tags were identified in either library. 'Germline tag < 9' are genes identified in the germline SAGE library, and either were not identified in the soma SAGE library or have a tag count ratio ≥ 2, but have a germline tag count < 9. '1 ≤ germline/soma < 2' refers to genes that were identified in both the germline and the soma SAGE libraries and have a germline/soma tag ratio ≥ 1 and < 2. The expanded category are genes that are only identified in the soma SAGE library, or genes that are identified in both the germline and the soma SAGE libraries with a soma/germline tag ratio > 1. **(C) **The corresponding microarray data for the germline-specific/enriched genes identified only by the SAGE. 'Microarray F.I.' refers to the adult/*glp-4 *fold induction value. 'Below confidence level' refers to genes with a 99% confidence level < 0. **(D) **Germline-specific or **(E) **germline-enriched genes identified by SAGE compared to germline-enriched genes identified by microarray plotted against the distribution of transcript levels based on tag counts.

We have identified 1063 genes whose transcripts are either enriched or specific in the germ line. We compared this list of genes to the list of germline-enriched genes in the adult obtained by microarray [11,12] to generate a more complete list of germline-enriched genes. Of the 1063 genes that we identified as being either germline-specific or enriched, 43.3% were also identified in the germline microarray analysis as being enriched in the germ line (This overlap is significant as compared to the overlap between the entire germline SAGE library and the microarray data; Fisher's exact p-value < 0.0001; Figure 5a) [11]. Therefore, we identified 603 genes as being germline-specific/enriched that were not identified by microarray. To determine how accurately SAGE identified germline-enriched genes, we analyzed the mRNA *in situ *expression patterns of these 603 genes using the NEXTDB database. 344 of the 603 genes have expression patterns in the NEXTDB database. Of these 344, 86% showed germline-specific/enriched expression and 11% show similar expression levels in the germ line and soma (Additional file 6). Only 10 genes (2.9%) showed soma-enriched expression (Additional file 6). Therefore, the large majority of the 603 genes that we identified as germline/specific enriched, but which were not identified as such by microarray, have mRNA *in situ *staining patterns consistent with our classification. We suspect that some of the genes not showing germline-enriched expression by *in situ *may actually be germline-enriched, but that we were unable to see a two-fold difference with the images provided in the NEXTDB database. Other genes may have been misclassified as germline-enriched by SAGE, although overall the SAGE classification correlates very well with the mRNA *in situ *expression patterns (Additional file 6).

The microarray analysis identified far more germline-enriched genes than SAGE in the adult hermaphrodite germ line (2304); only 20.0% germline-enriched genes identified in the microarray analysis were also identified as being germline-specific/enriched by SAGE (Figure 5a). To increase our understanding of why genes were identified in the microarray analysis but not SAGE, we looked at the 1844 germline-enriched genes in the microarray analysis that were not identified by SAGE. The majority (59.9%) of these genes were not identified by SAGE because they were expressed at too low of a level (Figure 5b); either no SAGE tags were detected (24.5%), or the germline SAGE tag count was below our threshold (≤ 9) and not counted in our analysis (35.4%). An additional 23.9% of genes could not be identified by SAGE because the genes do not have an associated SAGE tag in the germ line library (long SAGE) or soma library (short SAGE) (15.9%), or the tag was ambiguous (8.0%; tags that correspond to more than one gene; see Methods). 5.9% of the 1844 genes did have more tags in the germline SAGE library than the soma SAGE library, thus were germline-enriched, but were below our two-fold threshold, so were not included with the list of germline-enriched genes. Of the 1844 germline-enriched genes identified in the microarray analysis, 188 (10.2%) were identified to have higher transcripts level in soma than the germ line by SAGE. However, most of these had soma tag counts less than nine or had less than two-fold increase in tag counts; therefore, these genes do not meet our criteria to be confidently classified as tissue enriched. Only 25 genes (1.4%) were soma specific/enriched by SAGE (two fold more soma than germline tags with soma tag ≥ 9; Figure 5b). Therefore, there is very little discrepancy between the SAGE and microarray data; the vast majority of genes not identified in the SAGE (but germline-enriched by microarray), were not identified due to low levels of expression or the lack of unique SAGE tags associated with the genes. 200 genes were also randomly selected from the 1844 germline-enriched genes that were only identified by microarray and their *in situ *expression patterns we analyzed in NEXTDB. 85 of these 200 genes are included in the NEXTDB database and have discernable expression patterns. 77/85 have germline-specific/enriched expression patterns, suggesting that their identification as germline enriched in the microarray analysis is accurate.

### Germline-specific/enriched genes identified only by SAGE

SAGE identified 1063 germline-specific/enriched genes, but only 460 of these were also identified as such by the microarray analysis (Figure 5a) [11]. We further analyzed the 603 genes that were not identified by microarray to determine the potential reasons for only identifying them by SAGE (Additional file 6). Of the 603 genes, 64.0% were not identified in the microarray analysis either because no data was available for the gene on the microarray (34.0%; includes genes lacking a probe on the microarray), or the data obtained from the microarray was below the confidence level (30.0%) (Figure 5c). The remaining 217 genes (36.0%) gave ratios below two in the microarray analysis, suggesting that they are either enriched in the soma, or expressed exclusively in the soma (ratio of one). As an independent means of determining if these 217 genes are expressed at a higher level in germ line or soma, we analyzed their mRNA *in situ *expression patterns in the NEXTDB database [25]. 126 of the 217 genes are included in the NEXTDB database and have discernable expression patterns. Of these 126, 87% have an expression pattern that is either germline-specific or enriched, 10% show expression at similar levels in the germ line and soma, while only 2.3% show a higher level of expression in the soma than the germ line (Additional file 7). Therefore, the majority of genes identified by SAGE as being germline-specific/enriched, but which were identified as not enriched in the microarray analysis, do indeed show germline-specific/enriched expression patterns consistent with the SAGE classification.

To further investigate the cause of identifying genes as being germline-specific or enriched by one analysis (SAGE or microarray) and not the other, we determined if there was a correlation between gene expression level and the likelihood of being identified by both technological platforms. For this analysis, we compared the percentage of genes identified by SAGE that were also identified by microarray, relative to the number of tags identified for the genes. We divided the genes in this comparison into two groups; germline-specific genes (Figure 5d) and germline-enriched genes (Figure 5e), as determined by SAGE. Overall, genes that we labeled as germline-specific were more likely to be identified in the microarray data (65.2%) than genes that we labeled as germline-enriched (33.6%). Therefore, genes with little or no soma expression were more likely to be identified by both SAGE and microarray. Additionally, for germline-specific genes the percentage of genes identified by both platforms remained high, or even increased, as the expression level, based on tag count, increased. Surprisingly, this trend did not hold true for germline-enriched genes (Figure 5e). For germline-enriched genes (as determined by SAGE), a lower percentage of genes were identified as germline-enriched by microarray as the expression level (tag count) increased. Even though only 13% of the 60 SAGE identified germline-enriched genes with tag counts over 128 were also identified by microarray, 77% of those included in the NEXTDB *in situ *database (40/52) show germline-specific/enriched expression patterns; the other 23% show similar expression levels in germ line and soma. None appear to be enriched in the soma. Therefore, the SAGE classification of germline-enriched is consistent with the NEXTDB expression patterns for the majority of these highly expressed genes, even though few of these genes were identified by microarray as being germline-enriched.

### Chromosomal distribution of germline-expressed genes

**Figure 6:**
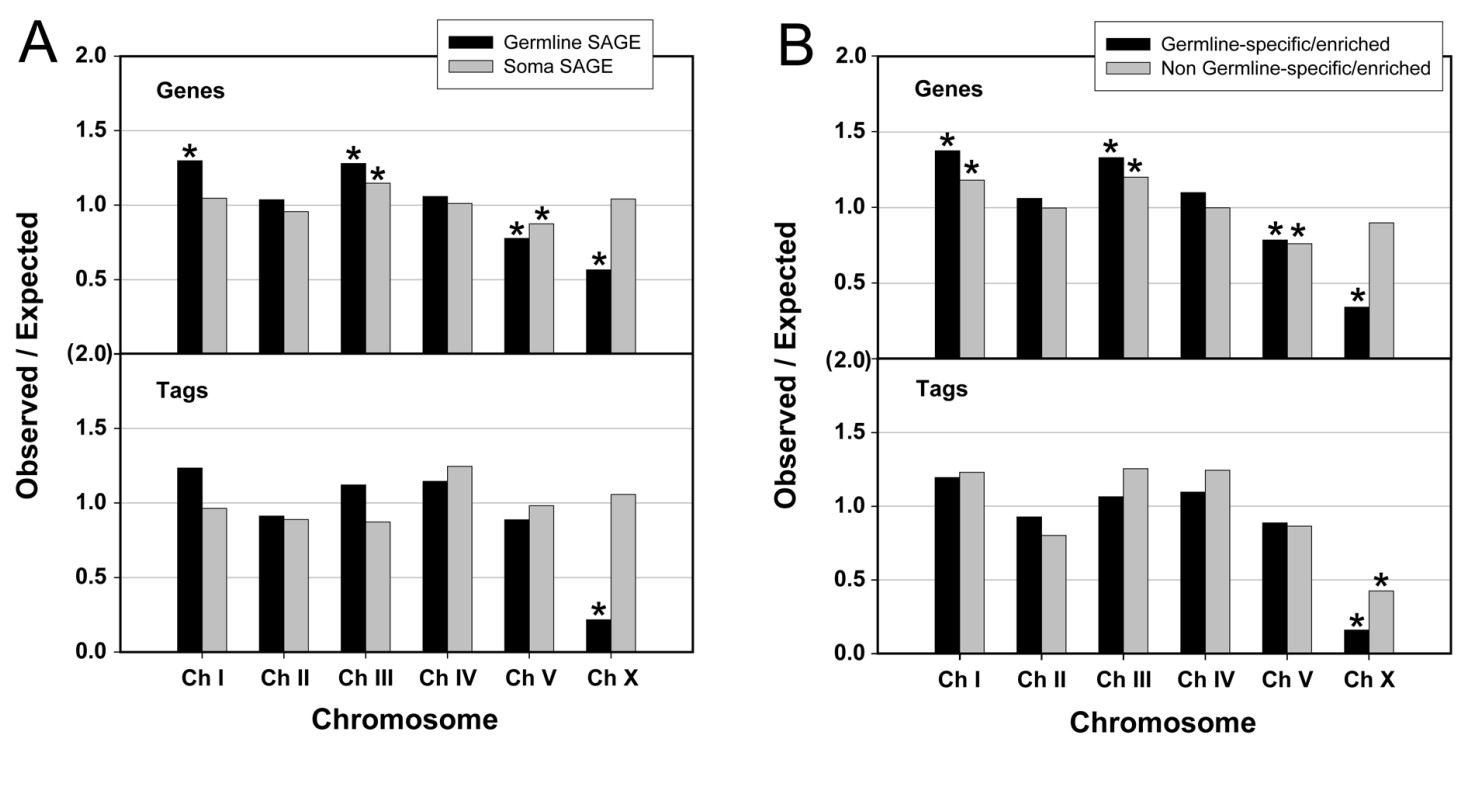
**Gene expression shows a biased chromosomal distribution**. Distribution was generated based on either the number of genes (A & B, upper panel) or the number of tags (A & B, lower panel). **(A) **The fraction of observed germline (black) and soma (gray) expressed genes compared to the expected value per chromosome is plotted (upper panel). The expected value is based on a random distribution of expressed genes throughout the genome as described in Methods. To compare expression levels of expressed genes on each chromosome (measured by the number of SAGE tags; lower panel), the average number of tags per gene in each gene set was first calculated then was multiplied by the number of genes observed in the same gene set for each chromosome to obtain the expected total number of tags (expression level) per chromosome. **(B) **Two sets of germline-expressed genes are compared; germline-expressed genes that are enriched or specifically expressed in the germ line (black), and germline-expressed genes that are expressed at a similar or higher level in the soma, as compared to the germ line (gray). The fraction of expressed genes compared to the expected value (upper panel), and the level of expression (lower panel), were determined as in (A). Statistical significance is marked by an asterisk (P < 0.001; hypergeometric probability test for the number of genes; re-sampling method for the number of tags, see Methods for details).

The microarray analyses of germline-enriched genes, described above, demonstrated a chromosomal bias for genes that are enriched for expression in the germ line [11,12,48]. It was found that genes with enriched germline expression are under-represented on chromosomes V and X, and over-represented on chromosomes I and III, based on an expected random distribution of expressed genes [11,12]. To determine if this chromosomal bias is only for germline-enriched genes, or if it also applies to germline-expressed genes that have similar or higher expression in the soma, as well as to determine if the level of expression is also chromosomally biased, we used our germline SAGE data to study the chromosomal distribution of germline-expressed genes. We first determined the chromosomal distribution of all 4699 germline-expressed genes, irrespective of their soma expression (Figure 6a). The chromosomal distribution of these genes is similar to the distribution determined by microarray analysis on germline-enriched genes [11,12]; germline-expressed genes are over-represented on chromosomes I and III, and under-represented on chromosomes V and X, assuming a random distribution of germline-expressed genes in the genome (Figure 6a). For comparison, we analyzed the chromosomal distribution of soma-expressed genes using the soma SAGE library data and found that the number of expressed genes for all chromosomes was similar to the expected value, except chromosomes III and V; soma expressed genes are over-represented on chromosome III and under-represented on chromosome V, although the observed numbers are closer to the expected values for the soma than the germ line. To determine if the chromosomal bias for soma and germline-expressed genes pertains only to the number of genes expressed on each chromosome, or if it also corresponds to expression levels of the expressed genes, we analyzed the number of SAGE tags for the genes expressed on each of the chromosomes. It was previously found that the average expression level of 258 oocyte-expressed genes was lower on the X chromosome than other chromosomes, based on microarray spot intensities [48]. By analyzing the number of SAGE tags for all 4699 germline-expressed genes, we found that genes on the X chromosome have a dramatic decrease in expression levels as compared to the other chromosomes (Figure 6a). In other words, not only are fewer germline-expressed genes found on the X chromosome than expected, but also the germline-expressed genes that are on the X chromosome are expressed at a lower level than expected. This differs from soma-expressed genes; both the number of genes and the expression levels of those genes are close to expected levels for the X chromosome.

To determine if the chromosomal bias of germline-expressed genes is limited to genes that are enriched in the germ line, or if it applies to all germline-expressed genes, even if they are also expressed at a similar or higher level in the soma, we split the germline-expressed genes into two groups; germline-expressed genes that are germline-specific/enriched, and germline-expressed genes that are expressed at a similar or higher level in the soma than the germ line (Figure 6b). We found that all chromosomes showed the same general trend for the number of genes expressed in the germ line on a given chromosome, whether they were germline-enriched or not, except for the X chromosome. Genes that are expressed at a higher level in the germ line than in the soma are less likely to be found on the X chromosome, while genes that are expressed at similar or higher levels in the soma than germ line are found on the X chromosome near the expected values, based on a random distribution (Figure 6b). However, both sets of germline-expressed genes, whether germline-enriched or not, are expressed at a lower level than expected (Figure 6b). It is interesting that the number of X chromosome genes expressed in the germ line is below the expected value for germline-enriched genes, but not for germline-expressed genes that are expressed in the soma at a similar or higher level (Figure 6b, top panel); both sets of genes are expressed at a lower level than expected (Figure 6b, lower panel). The lower level of X-chromosome genes expressed in the germ line is due to histone modifications, which leads to global silencing [48-50]. Germline-enriched genes presumably have functions that are more critical to the germ line; therefore, they may require higher expression that cannot be achieved on the X chromosome. Genes that are expressed in the soma and germ line may be more likely to be genes needed for embryogenesis. Since the X chromosome becomes reactivated as germ cells progress through oogenesis [48], germline-expressed genes that are not germline-enriched may be expressed in more mature germ cells as the X chromosome is becoming reactivated; therefore, this class of genes may not need to be excluded from the X chromosome.

## Discussion

We have identified 4699 genes that are expressed in the *C. elegans *adult hermaphrodite gonad (Additional file 1), which corresponds to roughly 1/5^th ^of all annotated *C. elegans *genes. We further show that 1063 of these germline-expressed genes have either enriched or specific expression in the germ line (Additional file 5). While 460 of these genes were previously identified as being germline-enriched [11], 603 are new to this classification (Additional file 6). These lists are an important resource for understanding the genes that are necessary for gamete formation and early embryogenesis. We also compared the data obtained from the microarray and SAGE platforms, identifying strengths and limitations of each. Finally, we added to our understanding of the chromosomal distribution of germline-expressed genes by including an analysis of expression levels, as well as including genes that are expressed in the germ line, but which are not enriched in the germ line. We found that, unlike genes that are germline-enriched, non-germline-enriched (but germline-expressed) genes are not under-represented on the X chromosome.

### High level of ribosomal gene expression

The class of genes with the highest representation in the germline SAGE library contains genes necessary for ribosomal formation and function (Additional file 2; Figure 3). These genes are expressed at a higher level in the germ line than soma (Figure 3b). Their high level of expression likely has to do with the amount of tissue generated in the adult germ line. In the adult soma, all tissues are fully formed and no further cell division occurs [9]. However, in the germ line, cell division continues throughout much of the life of the adult. In the distal end of the gonad, ~200–250 mitotic cells divide to replenish the cells that enter into meiotic prophase [3,51]. The cells that enter meiotic prophase progress through meiosis as they move proximally, finally culminating in surviving cells forming oocytes, which ovulate every ~23 minutes per gonad arm [4]. Along with a large amount of cell division occurring in the germ line, a significant amount of cell growth occurs. Ovulating oocytes have a final volume of ~20,000 um^3^, while cells in the distal end of the gonad are only a fraction of that volume [4]. Therefore, in the adult hermaphrodite germ line, a tremendous amount of cell division and cell growth is occurring. Indeed, the entire volume of the gonad is turned over every ~6.5 hours [2]. Tissue generation requires protein, and protein production requires ribosomes; therefore, it is logical that ribosomal genes are actively transcribed in a dividing tissue. Indeed, the correlation between cell growth and ribosome number was first observed in *E. coli*, in which it was found that the growth rate is proportional to the number of ribosomes [52]. The same correlation between growth rate and ribosome number has also been shown in a number of eukaryotic species [53,54].

### Comparison of SAGE and microarray technological platforms

By comparing our SAGE derived list of germline-enriched genes with the list generated by microarray [11], we have identified additional genes that are germline-enriched, adding confidence to the genes that were identified in both platforms, and have analyzed some of the strengths and weaknesses of each platform. Generally speaking, the degree of overlap between the SAGE generated list of enriched genes and the microarray list is relatively high. For the most part, the differences in the lists reflect the lack of data for certain genes in one of the analyses, primarily due to limitations of the platform, rather than conflicting data between the two analyses. For SAGE, far fewer germline-enriched genes were identified as compared to microarray; 1063 germline-enriched genes were identified by SAGE, while 2304 genes were identified by microarray (Figure 5a). For those genes that were identified in the microarray analysis, but not the SAGE, over 60% of the genes had few or no associated tags identified in the germline SAGE library, suggesting that expression levels were very low. Therefore, the cause of not identifying these genes may be that the sequencing depth was not sufficient for detection, and that greater sequencing depth may overcome this limitation. Newer sequencing technologies, which greatly increase the amount of sequence obtained, and at a much lower cost, will likely overcome this limitation. Indeed, recently prepared SAGE libraries are currently being sequenced at ~25× the depth of those in this study (millions of tags rather than hundreds of thousands; [55]; libraries of four million tags are now standard (DGM, unpublished results). Another limitation of SAGE, accounting for ~1/4 of the genes identified by microarray but not by SAGE, is that not all genes have unique SAGE tags; some genes lack the restriction site needed to generate a SAGE tag, while others generate SAGE tags that are not unique to a gene, and thus are ambiguous. The lack of a unique SAGE tag associated with some genes is an inherent limitation of SAGE and is difficult to overcome. However, similar problems exist for other technologies; for example, genes within a gene family may have similar sequence causing probes corresponding to more than one gene hybridizing to a single spot on a microarray. The use of Long SAGE rather than Short SAGE increases the probability that a given tag will be unique to single gene [46].

For those genes that were identified as being germline-enriched by SAGE, but not by microarray, over half were not detected by microarray because corresponding probes were not on the array, or because the data obtained for a given probe on the chip was deemed unreliable. Unlike SAGE, microarrays are a closed system that requires prior knowledge of genes; therefore, the microarrays are limited by the quality of gene annotation. The quality of the *C. elegans *annotation is very high; therefore, a similar analysis in a system with less extensive annotation would likely miss a higher percentage of genes.

The remaining ~48% of genes that were only identified by SAGE as being germline-enriched were detected by microarray analysis, but were determined to be expressed at an equal or lower level in the germ line as compared to the soma. We are confident that many of these genes, if not the majority, are germline-enriched because ~87% of these genes that are included in the NEXTDB database have staining patterns showing germline enrichment (Additional file 6). We are unsure as to why the microarray analysis did not identify these genes as being germline-enriched; however, we find it intriguing that the degree of overlap between the SAGE and microarray data for germline-enriched genes decreases as the expression level increases. Perhaps highly expressed genes, which would presumably have a significant level of labeled cDNA and a very bright hybridization signal on the microarray for both channels, could saturate the signal and prevent the difference in levels of expression from being detected [56].

This work has highlighted the benefit of using multiple technological platforms to generate a more complete list of the transcripts present in a given tissue. Although the amount of conflicting data between the two technologies was very low (Figure 5), the number of genes identified by only one of the technologies was large; ~56% of SAGE detected germline-enriched genes were not identified by microarray, and ~80% of microarray detected germline-enriched genes were not identified by SAGE.

### Other forms of regulation

Our germline SAGE library, and the microarray analyses [11,12], identified genes that are transcribed in the germ line. These analyses give a general idea as to the genes that are involved in gamete formation and early embryogenesis. However, it should be emphasized that transcription is only one level of regulation; the presence and relative abundance of mRNA transcripts may not always accurately reflect the function of the gene in the germ line. For example, post-translational control through phosphorylation regulates many aspects of germline development and function. Indeed, MAP kinase signaling, which presumably culminates in the phosphorylation of many protein targets, regulates at least eight distinct processes in *C. elegans *hermaphrodite germline development and function [57]. Translational control has also been implicated in multiple aspects of *C. elegans *germline development. For example, the proliferation vs. meiotic entry decision utilizes numerous proteins that regulate the translation and/or stability of target mRNAs [58,59]. The 3'UTR has been identified as the primary means by which the expression of germline-expressed genes is controlled [45]. We have found that mRNAs encoding RNA binding proteins are expressed at ~4× the level in the germ line than the soma. Therefore, the presence of an mRNA transcript does not fully predict a germline function. Indeed, some genes transcribed in the germ line may not function in the germ line at all, but rather are needed for embryogenesis. Simultaneous disruption of the activities of two translational regulators, GLD-1 and MEX-3, results in the formation of somatic tissues, such as muscles and neurons, in the germ line [60]. Presumably, genes necessary for the generation of these tissues are transcribed in the germ line; however, repression by GLD-1 and MEX-3 prevents the translation of these target mRNAs until the proteins are needed for embryogenesis. Therefore, the identification of genes transcribed in the germ line will need to be combined with protein expression and modification data to obtain a more complete picture of the factors necessary for proper germline function.

## Conclusion

Using SAGE we found that 4699 genes (~21% of all genes) are expressed in the *C. elegans *hermaphrodite germ line. A majority of the highest expressed genes are involved in translation, ribosome structure and biogenesis, and this general class of genes is expressed at a higher level in the germ line than the soma. Additionally, RNA binding proteins are expressed at a higher level in the germ line than the soma, suggesting that the control of gene expression through RNA metabolism is more predominant in the germ line than the soma. A comparison of germline-enriched genes identified through SAGE with a previously published list of germline-enriched genes identified by microarray found overlap with 460 genes, corroborating their classification as germ line enriched. Analysis of the genes identified by only one of the technological platforms identified potential strengths and weaknesses of each platform, as well as emphasized the importance of using more than one technological platform to obtain a more complete picture of global gene expression. Finally, the number of germline-enriched genes on the X chromosome is lower than that predicted assuming a normal distribution. However, the number of genes on the X chromosome that are expressed at a lower or equal level in the germ line than the soma is near what is expected.

## Methods

### Production and analysis of the germline SAGE library

Young adult wild type (N2) hermaphrodites grown at 20° were dissected ~18 hours after the fourth larval stage to isolate the gonad arms. Animals were dissected in 2 mL of PBS-EDTA-ATA (125 mM NaCl, 16.6 mM Na2HPO4, 8.4 mM NaH2PO4, 0.1 mM EDTA, 1 mM auxin tricarboxylic acid) with 0.2 mM Levamisole. Animals were dissected with two 25-gauge needles at the pharynx, allowing for the gonads and intestine to extrude from the body. The gonads were dissected away from the body by cutting at or near the spermatheca. ~150 gonad arms were placed in TRIZOL (Invitrogen, Carlsbad California) and the RNA was isolated following the manufacturers instructions. The germline SAGE library was prepared by standard methods as described in detail elsewhere [24,61,62]. Starting material was 143 ng of purified germline RNA and we used the established LongSAGE technique, which uses the enzyme *Mme*I to generate 21-bp tags [63]. The raw data for the germline SAGE library is deposited at , along with the data for other *C. elegans *SAGE libraries. SAGE tags were mapped to *C. elegans *genes using Wormbase WS160. Gene identification criteria used were: removal of duplicated ditags; resolve to lowest tag position; hide ambiguous tags; hide antisense tags; sequence quality > 99%; only coding RNA. Using these criteria we identified 92,007 tags in the germline SAGE library. We used the same criteria to analyze the soma SAGE library, which has been previously published [24]. From the soma library 91,888 tags were identified. For subsequent analysis, the total number of tags for each library was normalized to 100,000 tags, and only genes with a tag position 1 were used. If different unique tags were assigned to the same gene, both at position 1, the tag counts were combined to provide the total tag count for that gene. With these corrections, a total of 4699 and 5900 genes with unique tags were identified in the germline library and the soma library, respectively. There are 33 more genes identified in the soma library than previously reported [24]; this increase in the number of genes is likely due to the use of a more recent Wormbase freeze to map the tags to *C. elegans *genes (we used WS160 while the soma tags were originally mapped using WS140 [24]).

### Classification of RNA binding proteins

To identify proteins with putative RNA binding activity, we identified genes that encode proteins with a predicted RNA binding domain. Proteins were identified that had one or more of the RNA binding domains described previously [43]. We then filtered from this list, genes that encode proteins that are unlikely to have RNA binding function, such as those that are predicted to bind DNA, and genes encoding ribosomal proteins, using KOG classifications and other descriptions found in Wormbase freeze 190. Using these criteria we identified 319 putative RNA binding proteins (Additional file 3). 190 of these genes are expressed in the germ line, and 130 are expressed in the soma, based on the presence of one or more tags in the respective SAGE library. For this analysis, we wanted to obtain lists of RNA binding proteins, which could potentially be expressed in the germ line and in the soma, that are as complete as possible; therefore, in these lists we included genes that have one or more tags. Some of the RNA binding proteins that are associated with low tag counts may be background; for the entire germline SAGE library, ~25% of the genes are represented by just one tag.

### Identification of germline-specific/enriched genes

The germline and soma SAGE libraries were generated using the same anchoring enzyme, *Nla*III, but the tagging enzyme for each was different. The soma library was generated using the tagging enzyme *Bsm*FI, a 14 bp cutter, while in the construction of the germline library we used the tagging enzyme *Mme*I, a 21 bp cutter. This means the two libraries have tags of different lengths, which somewhat complicates a comparison between the libraries [46]. Since different tags are generated in long and short SAGE, genes may have a unique tag in one library but not the other. Therefore, in order to compare the germline and soma libraries to identify germline-specific genes, we excluded genes that have ambiguous tags with either short or long SAGE. We also removed genes that did not have assigned short SAGE tags. We defined "germline-enriched" genes as those that have associated SAGE tags in both the germline and the soma SAGE libraries, the germline tag count was ≥ 9 and there was at least a two-fold increase in the number of SAGE tags in the germline SAGE library as compared to the soma SAGE library. We defined "germline-specific" genes as those that have associated SAGE tags in the germline SAGE library, but not the soma SAGE library, and the number of tags in the germline library was ≥ 9. By using the ≥ 9 cut-off, we removed the majority of genes that failed to reach the p < 0.01 confidence level, which increases our confidence in the proper identification of germline-specific/enriched genes (Figure 4b). We chose a two-fold increase in SAGE tags based on a comparison with previously published microarray analysis of germline-expressed genes [11]. We compared genes that are found in both the microarray data and the SAGE data based on their fold increase. We compared the fold increase of genes that had at least two tags in both the soma and germline SAGE libraries with the fold increase of the same genes in the microarray data, in which a fold increase ≥ 2 was deemed to be germline-enriched [11]. We found that genes with 2 to 2.49 folds increase by SAGE had an average fold increase of 2.3 by microarray, and genes with 2.5 to 2.99 fold increase by SAGE had an average fold increase of 2.7 by microarray. Therefore, choosing a two-fold increase as the threshold for germline-enriched genes was roughly consistent with the microarray data. The distinction between germline-specific and germline-enriched is also likely dependent on the depth of sequencing of the SAGE libraries. It is likely that at least some genes with 0 tags in the soma SAGE library would have 1 or more tags if sequencing depth were increased.

### Classification of NEXTDB expression

Available expression patterns in NEXTDB were classified as follows: Class "I" = germ line is the only (obvious) site of expression in the adult worm; Class "II" = germ line is the major site of expression (with, say, > 70% of the expression intensity detected in the germ line – the 70% expression intensity level was determined by measuring the *in situ *staining intensity in the germ line, as compared to other tissues, of five randomly chosen Class II genes using Photoshop CS3 (Adobe); average 77.4% ± 5.4%; n = 5); Class "III" = germ line is one expression site among others in the adult worms (the expression intensity between germ line and soma is roughly the same); Class "IV" = gene is not expressed in the adult germ line; Class "?" = there is no staining, or expression pattern cannot be determined, usually because of weak signals; Class "-" = gene is not available in the NEXTDB database, or the *in situ *hybridization pattern is not available for the L4 – adult stage. Cross-referencing to the NEXTDB database was performed manually. Genes were searched in the NEXTDB database one at a time and assigned the corresponding *in situ *hybridization pattern according to the above classifications. Genes were searched in the NEXTDB, and the classifications assigned, by one person in order to minimize potential variation. The same individual blindly analyzed (not knowing previous classification) all *in situ *patterns a second time and determined a classification. <3% of classifications differed between the two replicates. Genes with different classifications were analyzed a third time to assign a final classification.

In addition to the genes identified in the germline SAGE library that we analyzed using the NEXTDB, we also randomly selected 1089 genes from the entire genome and classified accordingly using NEXTDB. 342 of these genes were in NEXTDB and showed a discernable *in situ *hybridization pattern. 54% of the 342 genes showed Class I or Class II expression patterns, while 46% showed Class III and Class IV expression pattern. Therefore, it is highly significant that 81% of the 124 genes (with discernable *in situ *pattern) that are highly represented in the germline SAGE library showed Class I or Class II expression patterns, as well as 86% of the 344 genes (with discernable *in situ *pattern) that are germline-specific/enriched only identified by SAGE showed Class I or Class II expression patterns (Fisher's exact p-value < 0.001).

### Chromosomal distribution of germline-expressed genes

To determine the expected number of expressed genes on a given chromosome, "SAGE tags available protein coding genes" were obtained from Wormbase  from the WS160 data freeze. For the germline SAGE library, only genes with associated Long-SAGE tags were used, while for the soma SAGE library, only genes with associated Short-SAGE tags were used. The fraction of genes from each chromosome with associated SAGE tags, as compared to the total number of genes in the entire genome with associated SAGE tags, was determined. For each chromosome, this fraction was multiplied by the total number of genes expressed in the germline or soma SAGE libraries; this provided the expected number of germline or soma expressed genes for each chromosome based on a random distribution of expressed genes. The observed number of genes expressed in the germline or soma SAGE libraries for each chromosome was then divided by the expected number of expressed genes per chromosome to obtain the observed/expected ratio that is plotted in the upper panel of figure 6a.

For comparing the observed versus expected expression levels for each chromosome, based on the number of tags, the expected value was determined by first calculating the average tags per expressed gene for the respective library (13.7 tags/gene for the germline SAGE library, 10.5 tags/gene for soma SAGE library). This average tags/gene value was then multiplied by the number of genes expressed on each chromosome from the corresponding library. This provided the expected number of SAGE tags expressed from each chromosome. The observed number of tags expressed was then divided by the expected number of tags expressed to obtain the observed/expected ratio that was plotted in the lower panel of figure 6a. Similar analyses were used on the germline-specific/enriched and the non-germline-specific/enriched datasets to obtain the observed/expected ratios that were plotted in figure 6b.

Statistical significance of the differences between the observed and the expected values was determined with a p-value < 0.001. For the comparisons based on the number of genes, hypergeometric probability tests were used [64]. For the comparisons based on the number of tags, a re-sampling approach was used. For example, in the case of chromosome I of the germline SAGE library, the corresponding number of tags of the 4694 germline SAGE genes was considered as the population, and the number of genes identified on chromosome I (1008), was considered as the sample size. Re-sampling was performed such that 1008 values were randomly picked from the population and then summed. The summed value corresponded to the total number of tags that was randomly determined. This procedure was performed 100,000 times to generate a probability distribution, which was used to obtain the p-value.

### SAGE and microarray comparison

We compared our SAGE generated list of germline-specific/enriched genes with the published list of microarray identified germline-enriched genes expressed in the adult [11]; we removed the 585 genes from the microarray data that only showed germline-enriched expression in the larvae because the SAGE data was obtained using adults. We analyzed the microarray generated germline-expressed data with WS160 Wormbase freeze genome annotations and updated gene IDs, removed non-protein coding genes, removed pseudogenes, removed retired genes and removed duplicated data entries. This resulted in an updated list of 2304 genes that were determined to be germline-enriched in the adult hermaphrodite by microarray. All 2304 genes have *wt*/*glp-4 *two-fold difference with a t-value over 99% confidence (p < 0.01). For genes with duplicated data entries in the microarray data, the average fold induction value was used. For the 603 germline-specific/enriched genes that were only identified by SAGE, the fold induction microarray data was kindly provided by Valerie Reinke (Yale University).

## Authors' contributions

DH isolated the gonadal tissue, contributed to the experimental design of the work and contributed to the data analysis. He also coordinated the study and drafted the manuscript. XW contributed to the experimental design of the work and performed the data analysis classifying genes into their KOG terms, classifying expression patterns from NEXTDB, classifying genes according to expression levels, comparing the microarray and SAGE data, determining the distribution of genes and tags in the genome and assisted in drafting the manuscript. YZ contributed to the construction and sequencing of the germline SAGE library. KW contributed to the initial bioinformatics analysis of the SAGE tags, posting and maintaining the tag library at Multisage. PE performed the statistical analysis of the chromosomal distribution of tags. DGM, SJJ, MAM, and RAH coordinated the construction and sequencing of the germline SAGE library. SJJ also critiqued the manuscript. DGM contributed to the experimental design of the work, and critiqued the manuscript. YK provided the *in situ *data in NEXTDB.

## Supplementary Material

Additional File 1 **All of the genes identified by germline SAGE library**. A list of genes that were identified by the germline SAGE library used for analysis. Click here for file

Additional File 2 **Set of 157 highly expressed genes identified in the germline library**. A list of genes, organized according to KOG classification, that have a tag count >60 in the germline SAGE library. Click here for file

Additional File 3 **Proteins with putative RNA binding activity**. A list of genes that were identified to encode proteins with putative RNA binding activity. Click here for file

Additional File 4 **Tag distribution of the RNA binding proteins identified in the germline and soma SAGE libraries**. Tag distribution of the RNA binding proteins identified in the germline and soma SAGE libraries. Proteins with potential RNA binding activity were identified as described in Methods. In total, 319 proteins were identified (Additional file 3), with 190 genes present in the germline SAGE library and 131 genes present in the soma SAGE library. Plotted is the total number of genes, of the 190 genes in the germ line and 131 genes in the soma, that have a given tag distribution. The number of genes in each tag range was determined and plotted against the tag distribution. Click here for file

Additional File 5 **The 1063 germline-specific/enriched genes identified by SAGE**. A list of genes that are identified to be germline-specific/enriched by comparing germline SAGE data with soma SAGE data. Click here for file

Additional File 6 **Set of 603 germline-specific/enriched genes identified only by SAGE**. A list of genes that are germline-specific/enriched genes identified only by SAGE as compared to microarray. Click here for file

Additional File 7 **Germline-specific/enriched genes identified only by SAGE with microarray F.I. < 2**. Summary of *in situ *hybridization expression pattern for those genes that are identified only by SAGE to be germline-specific/enriched and have a microarray F.I. < 2. Click here for file
