# Supplementary figures and images for: Identification of genes expressed in the hermaphrodite germ line of C. elegans using SAGE
Source: BMC Genomics. 2009 May 9;10:213. doi: 10.1186/1471-2164-10-213 (PMC2686737; doi:10.1186/1471-2164-10-213)

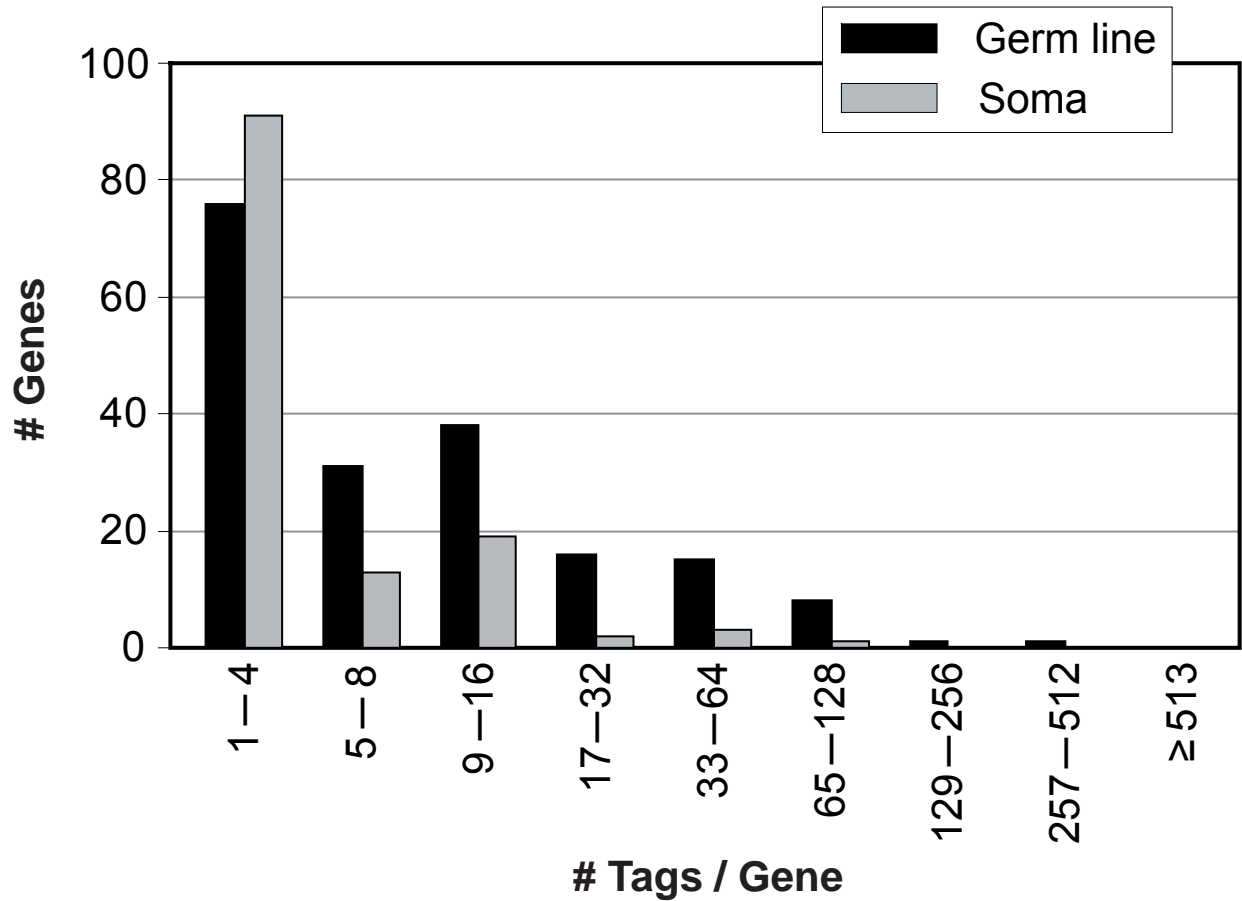

Supplement: Additional File 4 — Tag distribution of the RNA binding proteins identified in the germline and soma SAGE libraries. Tag distribution of the RNA binding proteins identified in the germline and soma SAGE libraries. Proteins with potential RNA binding activity were identified as described in Methods. In total, 319 proteins were identified (Additional file 3), with 190 genes present in the germline SAGE library and 131 genes present in the soma SAGE library. Plotted is the total number of genes, of the 190 genes in the germ line and 131 genes in the soma, that have a given tag distribution. The number of genes in each tag range was determined and plotted against the tag distribution. [file 1471-2164-10-213-S4.pdf]
