# Supplementary material for: Identification of genes expressed in the hermaphrodite germ line of C. elegans using SAGE
Source: BMC Genomics. 2009 May 9;10:213. doi: 10.1186/1471-2164-10-213 (PMC2686737; doi:10.1186/1471-2164-10-213)
Supplement: Additional File 7 — Germline-specific/enriched genes identified only by SAGE with microarray F.I. < 2. Summary of in situ hybridization expression pattern for those genes that are identified only by SAGE to be germline-specific/enriched and have a microarray F.I. < 2. [file 1471-2164-10-213-S7.doc]

**Additional file 7**

**Germline-specific/enriched genes identified only by SAGE with microarray F.I. < 2**

| Expression pattern identified in NEXTDB | No. of genes |
| --- | --- |
| Germline specific or enriched | 110 |
| Similar expression level in the germ line and soma | 13 |
| Higher level of expression in the soma | 3 |
| Expression pattern not available or cannot be determined | 90 |
